# Intelligibility of Haptic Signals in Vehicle Information Systems

**DOI:** 10.3390/s21134583

**Published:** 2021-07-04

**Authors:** Jong-Gyu Shin, Sang-Ho Kim

**Affiliations:** 1 Regional Industrial Management Research Institute, Kumoh National Institute of Technology, Gumi 39177, Korea; shinjg@kumoh.ac.kr; 2 Department of Industrial Engineering, Kumoh National Institute of Technology, Gumi 39177, Korea

**Keywords:** haptic, in-vehicle information system, signal intelligibility, Kansei engineering, driver’s response

## Abstract

Objective: The purpose of this study was to verify changes in a driver’s emotions through the physical characteristics of haptic signals. This is to improve the performance of drivers by designing haptic signals with emotional semantics. Background: Currently, drivers receive a variety of information through intelligent systems installed in their vehicles. Because this is mainly achieved through visual and auditory channels, an excessive amount of information is provided to drivers, which increases the amount of information and cognitive load that they must accept. This, in turn, can reduce driving safety. It is, therefore, necessary to develop a haptic signal, a sensory channel that has not been widely used in in-vehicle information systems. Methods: The experiment was performed to collect a driver’s emotions according to the haptic signal in a driving simulator. Haptic signals were designed by various frequencies and accelerations, and driver emotions were collected through Kansei engineering techniques and analyzed through factor analysis. To verify intelligibility, haptic signals were compared and evaluated based on response time, response rate, and amount of transmitted information. Results: The final determined emotional map consisted of dangerousness and urgency. Based on the emotional map, four emotional semantic haptic signals were designed. It was confirmed that these four signals displayed higher performance than the discriminability haptic signal in terms of response time, response rate, and amount of transmitted information. Conclusions: Using emotional maps, it is possible to design haptic signals that can be applied to various driving situations. These maps may also assist in securing design guidelines for haptic signals that apply to in-vehicle information systems.

## 1. Introduction

Haptic signaling is a field of research related to information transfer. It provides information through skin contact and occupies an important place in everyday human-computer interactions [1]. Haptic technology has recently been used for feedback or warning signals in various fields such as medical devices, automobiles, and musical instruments [2]. In particular, as automobiles develop advanced technologies such as autonomous driving, large volumes of information interact with drivers. However, most of these interactions depend on a visual interface, though some auditory interfaces are also used to assist the driver [3]. Current in-vehicle information systems (IVIS) require a breakthrough given the volume of information that will inevitably become more complex. A new interface using haptic signaling has been proposed to reduce dependence on visual and auditory interfaces. Wang et al. confirmed the efficiency of feedback through haptics for a lane-keeping assistant system [4], and Lv et al. suggested a more stable takeover method by using haptic signals in the takeover process of autonomous driving [5]. As such, haptics are utilized in interactions with the IVIS to reduce a driver’s cognitive load and improve stability. In addition, haptic signaling has emerged as a new interface to replace visual information that is lost in driving situations [6].

Haptic technology is widely regarded as a communication modality with the potential for recognition and expression of information because it accepts stimuli through the skin [6]. Additionally, haptics can only be implemented by easy parameter changes, and high accuracy is secured in recognizing signals [7]. In their research on tactile modality, Myles and Binseel argue that “tactile modality is a viable choice for the deliverance of information” [8]. However, because the information content is abstract and it interacts with simple human stimuli, a disadvantage of the haptic signal is that it is not intuitive [9]. To overcome this disadvantage, an information coding system that can easily learn and memorize haptics is needed. Moreover, if the semantics with the transmitted information are highly coded, the burden of processing information can be greatly reduced.

**Figure 1:**
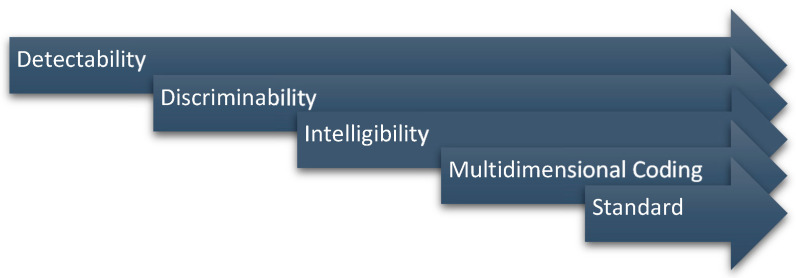
Good Information Coding System Framework.

Sanders and McCormick presented a framework for developing a good information coding system [10], which is shown in Figure 1.

Essentially, signals should be designed so that users can detect them and, when various types of signals are presented, users should be able to discriminate between them. Next, signals can reduce human cognitive loads by identifying semantics and ensuring the intelligibility of each signal. Signals with secured intelligibility should be able to be combined with signals from other sensory organs and standardized to make perfect signals. In the past, studies have been conducted to confirm the detectability of haptic signals, and they have shown them to be similar to auditory signals [11]. In terms of discriminability, it has been reported that a maximum of four haptic signals can be discriminated [12]. However, studies on securing haptic intelligibility have not been reported, and intelligibility must be ensured to reliably use haptics as signals for IVIS. In this study, an attempt was made to design the emotional semantic of a haptic signal to secure intelligibility; the Kansei Engineering technique was applied in this process. In addition, to verify the intelligibility of a haptic signal with an emotional semantic, a comparative evaluation was performed with the haptic signal that had secured discrimination.

## 2. Methods

### 2.1. Hypothesis

This study consisted of two hypotheses. The first was to prove that “it is possible to give emotional semantic meaning to haptic signals”. To prove this hypothesis, we identified an emotional change according to differences in the haptic signal design parameters and confirmed the appropriate semantic meaning. The second hypothesis is that “haptic signals with semantic meaning affect a driver’s cognitive enhancement”. This was tested to observe whether a semantic design could reduce a driver’s cognitive load.

### 2.2. Haptic Design

A haptic signal is generated through a device called an actuator or haptuator that generates vibrations. In this study, frequency and acceleration were specified as parameters for designing haptic signals. Acceleration can be adjusted by changing the voltage applied to the actuator, and frequency can be determined by adjusting the interval between signals as a parameter for how many signals are generated per second. The range of two independent parameters was designed to discriminate haptic signals within the frequency range of 80–250 Hz and the acceleration range of 2–5 G, according to previous studies [11,12]. For the haptic signal, the actuator Mark-II was selected by considering signal change range and a precision suitable for the research purposes. The Mark-II is a cuboid of 32 × 9 × 9 mm and it weighs only 9.5 g; however, it can transmit various signals because it can load a frequency range of 90–1000 Hz and has a voltage of a maximum of 3 V. Electrical signal characteristics applied to the Mark-II were controlled using NI Labview software and a D/A converter.

### 2.3. Subjects

The subjects were 20 university students and graduate students with driver’s licenses. The average age was 26.6 years old and the average driving experience was 4.9 years. Before the experiment, sufficient practice time was provided to adapt to the simulator, and the experiment was conducted when an understanding of its processes was sufficiently secured. To control external factors affecting the performance of the subjects, the experiment was conducted with individuals who had no health problems, did not drink alcohol the day before, and had sufficient rest.

### 2.4. Data Gathering

The data measured in this study consisted of three types (emotional questionnaire data, response rate, and response time). Emotional questionnaire data were collected to confirm the emotional information given by the haptic signal to drivers. The questionnaire was produced using the Kansei engineering technique. The Kansei engineering technique is quantitatively used to identify emotional semantics [13]. Kansei engineering can measure emotions using Kansei words (KWs). A KW is a word that expresses emotion and is mainly used as an adjective. These words were derived using the semantic differential method. Osgood et al. developed the semantic differential method as an application of Osgood’s more general attempt to measure the semantics or meanings of words, particularly adjectives and their referent concepts [14]. Emotions generated from various haptic signals were measured with 20 KWs on a 5-point Likert scale. Each KW was collected as a pair of adjectives related to a semantic meaning that should be defined as a warning signal by referring to the KWs used in a related study [15,16,17].

In addition, to verify the usefulness of the semantic design, the response rate (RR) and response time (RT) to the haptic signals given in the driving situation were designated as dependent variables. RR and RT were used to compare haptic signals among the groups (emotional semantic design and just discriminability). The ratio of a driver’s correct response between the two signal groups was designated as RR, and the time it took to decide was designated as RT.

### 2.5. Experimental Procedure

**Figure 2:**
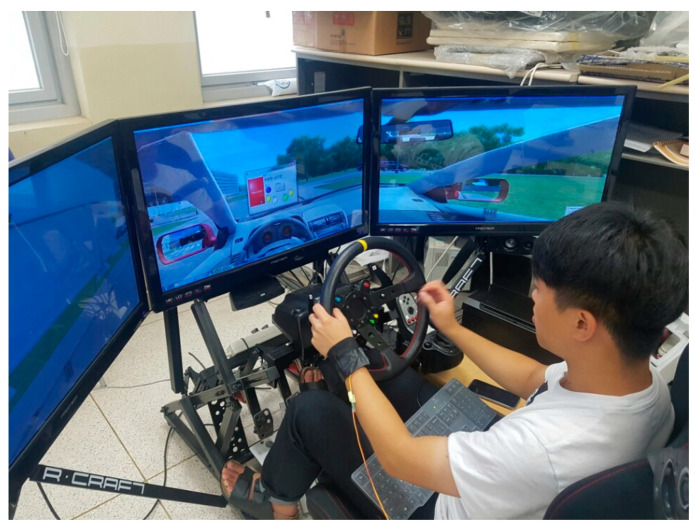
Experimental environment.

The study was conducted in a simulation environment, as shown in Figure 2. Two types of experiments were conducted to verify the hypothesis.

**Figure 3:**
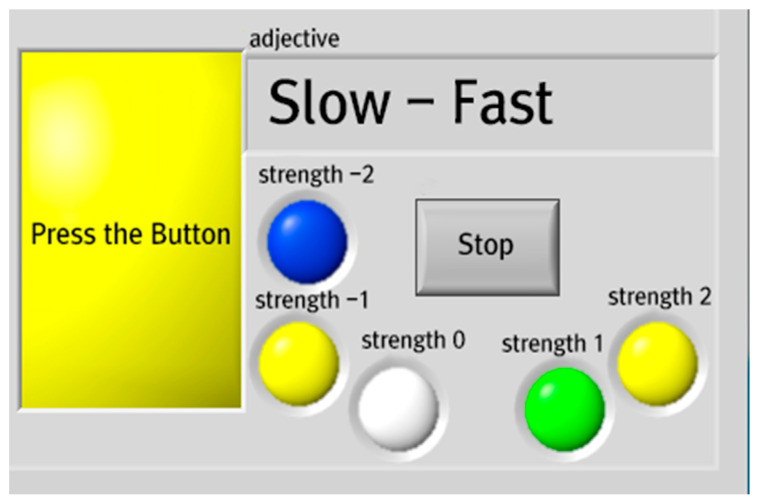
Interface for KW measurement.

The experiment proceeded in two stages. First, to confirm the first hypothesis, an emotional evaluation using the Kansei engineering method was performed. Subjects gave scores for KW displayed on the screen through buttons on the steering wheel, as shown in Figure 3. The scores were related to the haptic signals that randomly appeared while driving.

Four haptic signals were presented per experiment, and scores for 20 KWs were measured through 20 repetitions.

To confirm the second hypothesis, a comparative evaluation of the haptic signal group was performed. For the same 20 subjects, the experiment was repeated 5 times for 4 signals with only discriminability. The same experiment was performed for 4 signals with semantic design to measure the RR and RT between the two haptic signal groups. RR proceeds by pressing the button of the same color provided on the steering wheel with respect to the color that appears on the left side of the screen in Figure 3. If the subject presses a button of a different color, the answer is incorrect. As in the first experiment, the second experiment proceeds after the recognition of a haptic signal that is randomly presented while driving. Considering that the warning signal should induce the driver to react quickly in a driving situation, the RT measurement was set so that the subject could respond within 3 s. Therefore, if the subject took longer than 3 s to respond after the presentation of a haptic signal, we determined that their response was not performed.

### 2.6. Analytical Methodology

In this study, exploratory factor analysis (EFA) was used to statistically analyze emotional questionnaire data. All analyses were conducted using Minitab 18. EFA serves to identify potential structures based on the correlations between measured variables [18]. EFA was used to derive KW clusters with commonality based on the correlation between KW scores. Based on the analyzed KW score, an emotional map was derived to understand the emotional semantic meaning of the haptic signals. By testing independence with a chi-square analysis, RR analyzes whether there is a difference in response accuracy between the two haptic signal groups. To minimize individual differences between subjects, RT performed a designed ANOVA analysis on the subjects; this also helped to determine whether there was a difference between the two signals. In addition, the amount of transmitted information was analyzed with the use of information theory [19]. We established how much information from two types of the haptic signal group could be delivered to a given driver.

## 3. Results

### 3.1. Emotional Map for Haptic Signals

To confirm the first hypothesis, an emotional evaluation was performed on the haptic signal through KW. In the haptic signal design, the two parameters (frequency, acceleration) were independent of each other, and the detection range was confirmed through prior research [11]. In addition, because a previous study conducted work in which the driver discriminated between four signals at once [12], the experiment was configured so that four different signals were presented in one block. Table 1 shows the parameter sets of the determined haptic signals.

Among driving tasks, the first set of haptic signals in Table 1 was presented, and emotional scores were collected by using KWs in Table 2. A total of three experiments were performed up to Set 3 for the same KWs.

As a result of analyzing the emotional score data through factor analysis, it was found that the emotions for the haptic signals were gathered on two axes in the scree plot. The KWs included in the factors are shown in Table 3 below.

In Table 3, the name of the factor axis was determined based on the KWs belonging to the two axes. In the case of factor 1, it was called “urgency”, as KWs expressing sensitivity to the intensity or response speed of a warning situation were induced according to the characteristics of a given haptic signal.

Factor 2 comprised the KWs for detection of a signal, and its axis was called “awareness”.

**Figure 4:**
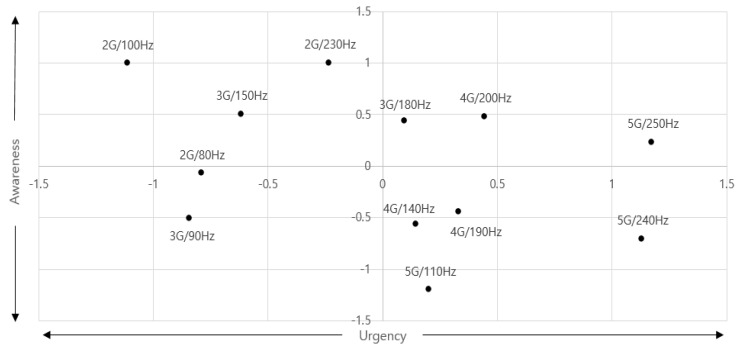
Emotional map of “urgency” and “awareness”.

**Figure 5:**
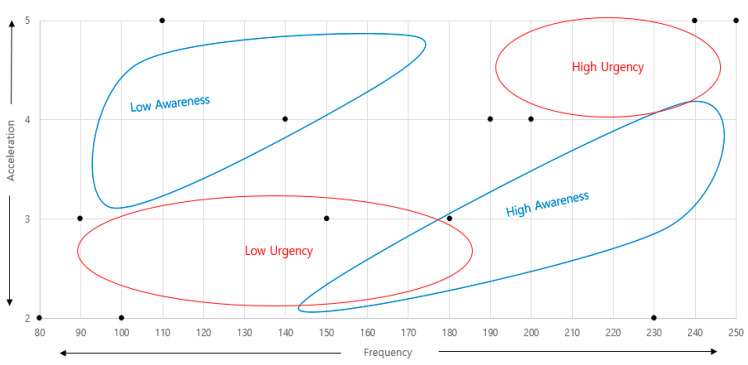
Emotional map of “frequency” and “acceleration”.

The result of expressing the haptic signals on the emotional axes of urgency and awareness is shown in Figure 4. In Figure 4, urgency was generally felt in the high acceleration and high-frequency bands. Awareness had an emotional semantic meaning in the low-acceleration and high-frequency bands. To make it easier to represent, the emotion map was rewritten by converting it to the frequency and acceleration axes. It can be seen in Figure 5 below.

In Figure 5, when designing a haptic signal with emotional semantics, changes in design parameters can be confirmed. If a haptic signal that has a large meaning for urgency is needed, it should be designed with high acceleration in the high-frequency band, and it should also take into account a low acceleration range and a high-frequency range for awareness. However, awareness is a characteristic that must be possessed as a signal, and signals without awareness cannot be used in IVIS. Therefore, the evaluation was once again conducted within the design range of awareness.

New haptic signal sets were designed to include all parameters, as in the previous experiment that used a frequency of 140–250 Hz and an acceleration characteristic of 2–5 G. These were the ranges of awareness identified through the first emotional map, and they are shown in Table 4.

For KWs, 20 pairs were newly extracted through a significance test, except for adjectives indicating awareness (Table 5).

As a result of checking the scree plot in the second evaluation, it was found that it was compressed into two axes, and the appropriate KW group was identified, as shown in Table 6.

**Figure 6:**
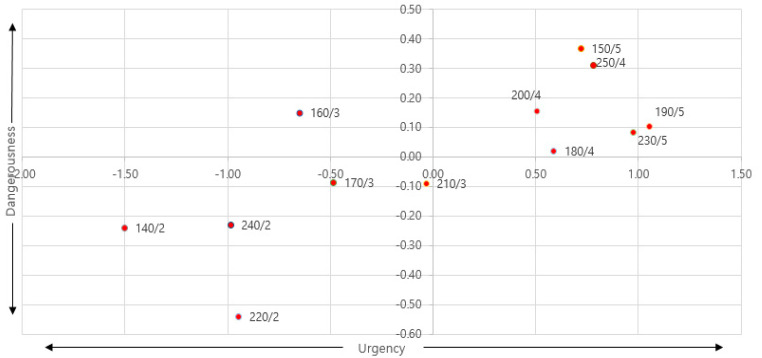
Emotional map of “urgency” and “dangerousness”.

In Table 6, the KWs included in the factors were identified, and a new emotional axis was named. Factor 1 comprised KWs that express sensitivity to the intensity or response speed of a warning situation induced according to the characteristics of a given haptic signal. Therefore, it was called “urgency”. Factor 2 comprised KWs that express a degree of tension or warning that is felt in the signal, and it is was named “dangerousness”. If the haptic signal was expressed on the set urgency and dangerousness axes, it is shown in Figure 6.

**Figure 7:**
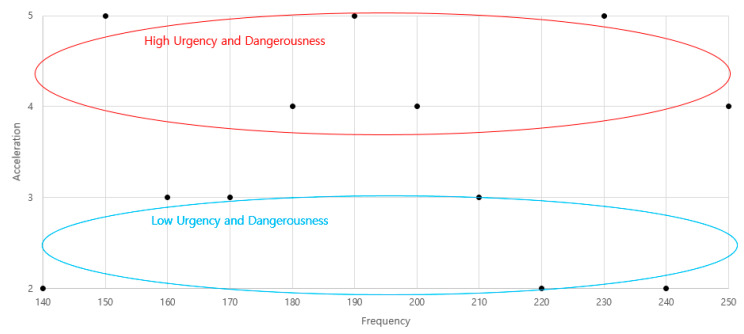
Emotional map of “frequency” and “acceleration”.

As a result of mapping haptic signals for urgency and dangerousness, we confirmed that, the lower the acceleration, the lower the urgency and dangerousness. When the map was re-created based on frequency and acceleration, which were design parameters of the haptic signal, the change in emotion could be seen more clearly, as shown in Figure 7. It can be seen that the emotional semantics of urgency and dangerousness were greatly affected by acceleration within the range of 140–250 Hz, which is the frequency range in which awareness is secured.

Based on this result, it was confirmed that it is possible to design a haptic signal that can give different meanings in terms of urgency and dangerousness by changing frequency and acceleration, i.e., the main design parameters of the haptic signal. In addition, it can be seen that it is appropriate to design acceleration of 3 G or less between the frequency range of 140–250 Hz when information is transmitted in a situation of low dangerousness or low urgency through a haptic signal. On the other hand, designing an acceleration of 4 G or higher in the frequency range of 140–250 Hz is preferable when transmitting a warning signal in a situation where there is significant dangerousness and an urgent response is required.

### 3.2. Verification of Emotional Semantic Haptic Signals

In designing haptic signals with emotional semantics by using the previously derived emotional map, a verification experiment was conducted to check whether a driver’s cognitive load could be reduced by increasing intelligibility. In our examination of the semantics of detailed haptic signals by frequency, we observed no significant differences in dangerousness and urgency; however, a difference in emotional semantics was confirmed, and four signals with different meanings were extracted. This is shown in Table 7.

A comparative experiment was conducted to establish whether the emotional meaning design of the haptic signal shown in Table 7 could reduce a driver’s cognitive load. Before the experiment, the subject was sufficiently trained to understand the discriminability and emotional semantics according to the haptic signals. The collected data were analyzed in terms of response rate, response time, and delivered information content of the two haptic signal groups.

**Figure 8:**
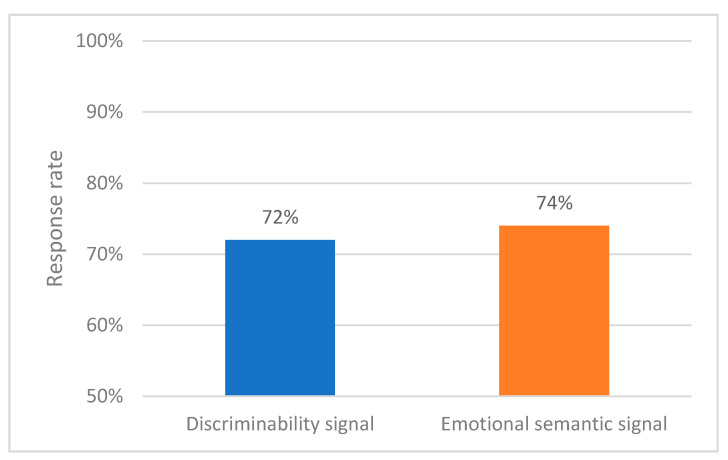
Response rate (discriminability vs. emotional semantics).

First, if there was confusion in the semantic design due to the problem of accuracy in relation to how the button set in the haptic signal is pressed after the signal is presented, RR would show a lower level than the discriminability signal group. However, as a result of the chi-square independence test shown in Figure 8, the discriminability signal group showed an average response of 72%, and the emotional semantic signal group showed an average response of 74%, which is 2% more.

It is unclear whether RR responds to signals more accurately by designing emotional semantic signals (Table 8). RR is the result of a user’s decision-making and response selection to the stimulus. Signal selection implemented in this process can secure sufficient accuracy if discriminability is secured. Both signal groups used in the experiment showed no significant difference in RR because they were signal groups with secured discriminability.

Next, a *t*-test was used for comparative analysis to see whether there was a difference in response time between the two groups of haptic signals. Response time is the time it takes for a driver to respond to the haptic signal. If the time to move the body is the same, the remaining time can be viewed as the information processing time of the brain. Therefore, if the emotional semantic design of haptic signals improved cognition, it was expected that the response time would decrease as the brain’s information processing time decreased. Response time was analyzed, except for haptic signals that did not respond.

**Figure 9:**
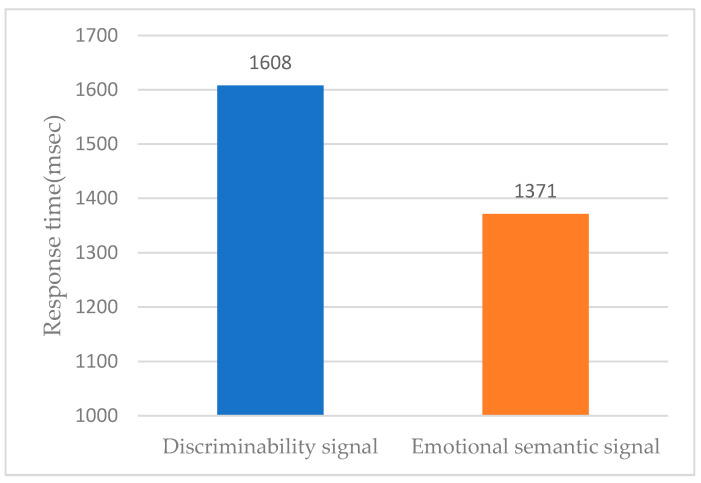
Response time (discriminability vs. emotional semantics).

An analysis of differences in average response time between the two groups found that the discriminability signal group average was 1608 ms and the emotional semantic signal group average was 1371 msec (Figure 9). The emotional semantic signal group showed a 237 ms faster response than the discriminability signal group. Additionally, as shown in Table 9, there was a difference in the response times of the two groups at a significance level of 5% (*p*-value: 0.000).

**Figure 10:**
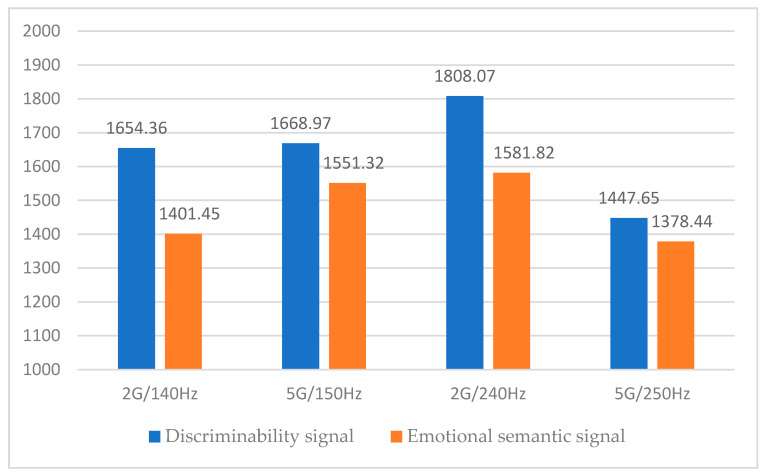
ANOVA results for two haptic signal groups.

Because there was a difference between the two signal groups, a within-subject design ANOVA was performed to establish which part of the signals differed within each signal group. Table 10 and Table 11 and Figure 10 show the ANOVA results.

After confirming the difference between the discriminability signal and emotional semantic signal through ANOVA, a significant difference was found at a significance level of 5%. In addition, the haptic signal of the emotional semantic design showed a faster response time (Figure 10), and it was once again confirmed that the emotional semantic design, created according to the parameters of the haptic signal, helped improve usability from the driver’s perspective.

Finally, to confirm whether the haptic signal designed for emotional meaning improved information transmission ability, the amount of transmitted information from the two signal groups was obtained and a comparative analysis was performed. Table 12 shows how the subjects responded to the four haptic signals to calculate the amount of transmitted information.

Analysis of the number of correct answers for each signal in Table 12 found that the number of correct responses to the 5 G/250 Hz signal was 83 in both signal groups, which was the highest number compared to the other signals for which the results were the same. For 2 G/140 Hz and 2 G/240 Hz, the number of correct answers for each signal was relatively high in the emotional semantic signal group.

Table 13 shows the result of calculating the amount of transmitted information for the haptic signal group according to information theory. As a result of the *t*-test at a significance level of 0.05 for the two signal groups, the *p*-values of each group were 0.005, 0.008, and 0.012 in relation to transmitted information, equivocation, and noise. These values were all statistically significant.

**Figure 11:**
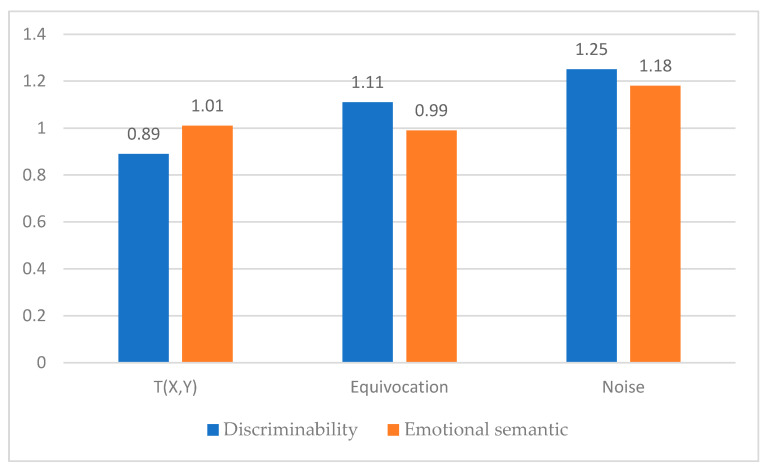
Differences between the two signal groups based on information theory.

As can be seen in Figure 11, the amount of transmitted information in the emotional semantic signal group was 1.01, which was higher than that in the discriminability signal group, which was 0.89. For the discriminability signal and emotional semantic signal groups, equivocation was 1.11 and 0.99, respectively, and noise was 1.25 and 1.18, respectively; these values were relatively small for the semantic signal group.

Therefore, it could be determined that the emotional semantic haptic signal with a large amount of transmitted information and low equivocation and noise transmitted information more effectively and clearly.

## 4. Discussion

In this paper, the use of the skin channel, which is a new sensory modality, was proposed for in-vehicle information systems (IVIS). Accordingly, we attempted to secure intelligibility through the design of emotional semantics related to haptic signals accepted by the skin channel. Warning signals need to be provided to users in response to situations of urgency and danger and, in this study, urgency, awareness, and dangerousness differed as the level of design parameters changed. This proves that it is possible to design emotional semantic haptic signals according to driving situations. In addition, it was confirmed that the designed emotional semantic signal provides faster feedback to the driver than the discriminability signal. This confirms that it is possible to design an emotional semantic that can use a haptic signal as a warning signal, as well as one that can convey emotion.

The haptic signal was supposed to respond within 3 s. In the experimental results, the emotional semantic signals showed a response time of up to 1.5 s. This is the same as reacting after moving about 41 m (1.5 s) in a vehicle running at 100 km while maintaining a 100 m inter-vehicle distance. As such, studies have been conducted in advance in relation to usability and safety [3,20]. However, this study evaluated a driver’s detailed sensibility and usability together and presented a haptic design range that could respond more quickly and accurately to a user. Additionally, Ji et al. (2011) set the response within 5 s as the standard [3], but this study confirmed the response within 3 s, meaning our study is more advantageous in terms of securing safety.

The results of the study identified several considerations required when designing haptic signals. First, it can be seen that a signal with low acceleration in a low-frequency band should not be used as a signal because it is not suitable for securing awareness. Additionally, it is more effective to design haptic signals through a change in acceleration within a range in which awareness is secured. Compared to the signal without an emotional semantic design, the haptic signal with the emotional semantic design was able to confirm improvements in cognition through the response rate and response time of a given driver. As a result of comparing the amount of transmitted information, equivocation, and noise of the two signal groups, the semantically designed signal showed a higher amount of transmitted information, lower equivocation, and noise compared to the signal with discriminability. This can be interpreted as a result of securing the intelligibility of a haptic signal through the design of an emotional semantic signal. Petermeijer et al. suggested that, when haptic signals were applied to the IVIS, response time was reduced; however, this could cause significant annoyance [21]. To solve this problem, it is necessary to perform more detailed semantic coding to induce positive emotions in a driver. In addition, measurement variables such as known biosignals and facial expressions can be used to measure emotion. This allows one to see a driver’s emotions more clearly.

The emotional engineering technique used in this study is also used to develop emotional robots in the field of human–robot interaction [22], and to evaluate emotions in voice-based human–AI interaction [23]. The autonomous vehicle we seek to develop will actively interact with its driver based on the multimodal interface [24], and haptic research and development will need to be conducted accordingly. Particularly in terms of semi-autonomous driving, the effect of a visual display, such as a HUD, would be appropriate, as drivers look straight ahead [25]. However, in autonomous driving, signals must be presented according to a driver’s state (sleep, watching a movie, reading, etc.) and, in some states (drowsiness, sleep), a haptic signal may be more effective than a visual interface.

This study has limitations, such as the fact that it only targeted subjects in their 20s. Therefore, we judge that it is difficult to generalize the experimental results to a more diverse and wider range of groups. To generalize our results, it is necessary to study another group (considering the human factor) of human subjects.

In addition, because the experiment was conducted using a virtual simulator, accidents did not pose a direct threat to drivers. This fact can potentially induce a comfortable attitude in the subjects and influence how their intelligibility is improved in dangerous situations through haptic signals. Therefore, to control this limitation, an additional evaluation based on an actual vehicle needs to be made.

## 5. Conclusions

Based on the emotional map confirmed through this study, it is possible to design a haptic signal suitable for the level of an appropriate situation, such as an urgent situation. IVIS application using auditory and haptic modality is required in situations where visual IVIS is not available, such as drowsy driving or non-driving tasks. In addition, to be applied to autonomous driving technology, it is necessary to consider driving environment scenarios (curves, obstacle avoidance, lane changes, etc.) in human-out-of-the-loop situations [26,27]. Therefore, IVIS research using haptic signals should be conducted as a guideline or standard stage rather than a basic stage, and this research can be supported. In the future, it is necessary to establish intelligibility in special circumstances, such as a takeover situation in autonomous driving or drowsy driving. Through this, it is expected that we will be able to derive advanced IVIS design guidelines for haptic signals.

## Figures and Tables

**Table 1:** Haptic signal sets in the experiment.

Set 1	Set 2	Set 3
2 G/80 Hz	2 G/100 Hz	2 G/230 Hz
3 G/90 Hz	3 G/150 Hz	3 G/180 Hz
4 G/200 Hz	4 G/190 Hz	4 G/140 Hz
5 G/250 Hz	5 G/240 Hz	5 G/110 Hz

**Table 2:** Kansei words used in the experiment.

Relaxed–Emergent	Calm–Terrified
Negligible–Attentive	Tender–Harsh
Thin–Bold	Light–Heavy
Ambiguous–Distinct	Probable–Certain
Leisurely–Pressing	Ordinary–Salient
Safe–Dangerous	Minor–Critical
Slight–Chunky	Low–High
Vague–Clear	Subsidiary–Essential
Trivial–Significant	Declining–Rising
Mild–Strong	Simple–Complex

**Table 3:** KWs strongly associated with reduced factors.

Factor 1	Factor 2
Mild–Strong Low–High Leisurely–Pressing Simple–Complex Tender–Harsh Negligible–Attentive	Ordinary–Salient Subsidiary–Essential Vague–Clear Ambiguous–Distinct

**Table 4:** New haptic signal settings in the experiment.

Set 1	Set 2	Set 3
2 G/140 Hz	2 G/240 Hz	2 G/220 Hz
3 G/170 Hz	3 G/210 Hz	3 G/160 Hz
4 G/200 Hz	4 G/180 Hz	4 G/250 Hz
5 G/230 Hz	5 G/150 Hz	5 G/190 Hz

**Table 5:** Kansei words used in the second experiment.

Static–Dynamic	Loose–Tight
Laid-back–Tense	Safe–Dangerous
Ordinary–Special	Quiet–Flush up
Calm–Panic	Weak–Strong
Easygoing–Excited	Carefree–Anxious
Minor–Major	Insensitive–Sensitive
Comfortable–Disturbed	Peaceful–Emergent
Relaxed–Nervous	Careless–Cautious
Leisurely–Urgent	Unstable–Stable
Mild–Serious	Frivolous–Prudent

**Table 6:** Second KWs strongly associated with reduced factors.

Factor 1	Factor 2
Static–Dynamic Laid-back–Tense Ordinary–Special Calm–Panic Easygoing–Excited Minor–Major Comfortable–Disturbed Relaxed–Nervous Leisurely–Urgent Mild–Serious Safe–Dangerous Loose–Tight	Unstable–Stable Careless–Cautious Frivolous–Prudent

**Table 7:** Emotional semantic design haptic signals and discriminability haptic signals.

Haptic Signals (Acc/Freq)	Emotional Semantics	Discriminability
2 G/140 Hz	Urgency low ↓ Dangerousness high ↑	No. 1 haptic signal
5 G/150 Hz	Urgency high ↑ Dangerousness low ↓	No. 2 haptic signal
2 G/240 HZ	Urgency low ↓ Dangerousness low ↓	No. 3 haptic signal
5 G/250 Hz	Urgency high ↑ Dangerousness high↑	No. 4 haptic signal

**Table 8:** Chi-square analysis results for response rate.

	Discriminability	Emotional Semantics	Total
Response	288	296	584
Non-response	112	104	216
Total	400	400	800

Pearson chi-square = 0.406; DF = 1; *p*-value = 0.524.

**Table 9:** *t*-test analysis results for response time.

Haptic Signals	Sample Size	Average	S.D.	S.E
Discriminability	288	1608	470	28
Emotional Semantic	296	1371	499	29

*t*-value = 5.89, *p*-value = 0.000.

**Table 10:** ANOVA results for discriminability signal group.

	S.S	DF	M.S	F-Value	*p*-Value
Discri. signals	66,202,81	3	220,670	12.65	0.000
Subjects	27,160,692	99	274,350	1.57	
Signal X Subject	51,820,671	297	174,480		
Error		0			
Total	85,601,644	399			

**Table 11:** ANOVA results for emotional semantic signal group.

	S.S	DF	M.S	F-Value	*p*-Value
Semantic signals	3,192,551	3	1,064,184	3.67	0.013
Subjects	23,080,858	99	233,140	0.80	
Signal X Subject	86,027,400	297	289655		
Error		0			
Total	112,300,809	399			

**Table 12:** Accuracy by haptic signals.

Signals		2 G/140 Hz	5 G/150 Hz	2 G/240 Hz	5 G/250 Hz	No-Response
		(Correct Response Number/Total Signal Number)				
Discriminability	2 G/140 Hz	69/100	5/100	19/100	1/100	6/100
	5 G/150 Hz	9/100	73/100	12/100	5/100	1/100
	2 G/240 Hz	24/100	8/100	63/100	1/100	4/100
	5 G/250 Hz	1/100	11/100	3/100	83/100	2/100
Emotional semantic	2 G/140 Hz	73/100	1/100	18/100	0/100	8/100
	5 G/150 Hz	18/100	70/100	4/100	3/100	5/100
	2 G/240 Hz	7/100	19/100	70/100	1/100	3/100
	5 G/250 Hz	6/100	6/100	0/100	83/100	5/100

**Table 13:** Results of information theory-based analysis of the two haptic signal groups.

	H(X)	H(Y)	H(X,Y)	T(X,Y)	Equivocation	Noise
Discriminability	2.00	2.14	3.25	0.89	1.11	1.25
Emotional semantic	2.00	2.19	3.18	1.01	0.99	1.18
*p*-value				0.005	0.008	0.012

## Data Availability

The data presented in this study are available on request from the corresponding author. The data are not publicly available due to privacy.
